# Healing Outside of a Doctor’s Office: Advancing Reproductive Wellness Through Storytelling in the Dobbs Era

**DOI:** 10.1177/24731242251372699

**Published:** 2025-08-25

**Authors:** Robyn B. Adams, Stacie McCormick, Amber T.A. Reid, D’Andra Willis, Qiana Arnold, Helen Zimba, Tambra Morrison, Marsha Jones

**Affiliations:** ^1^ Department of Advertising & Brand Strategy, Texas Tech University, Lubbock, Texas, USA.; ^2^ Department of English, Comparative Race & Ethnic Studies, Women & Gender Studies, Texas Christian University, Fort Worth, Texas, USA.; ^3^ The Afiya Center, Dallas, Texas, USA.; ^4^ The Afiya Center, Reproductive Justice Organization, Dallas, Texas, USA.

**Keywords:** Reproductive Justice, Black, abortion, health care, Black feminism

## Abstract

Amber Thurman, Candi Miller, Porsha Ngumezi, Josseli Barnica, and Neveah Crain highlight the tragic outcomes of restrictive abortion bans post-Dobbs v. Jackson Women’s Health Organization. Their stories underscore the need to shift away from viewing medical institutions as the only sources of reproductive care. The Afiya Center’s Livable Black Futures Collective advocates for community-based knowledge through a Reproductive Justice storytelling framework. By emphasizing the injustices faced by Black women and birthing people, these narratives aim to reclaim bodily autonomy, dismantle stigma, and foster resilience, ultimately serving as a powerful catalyst for collective healing and activism against systemic oppression.

For true, from the long end

of the looking glass, it appears

isolation is one way to quell

a revolution.^1^ (Hill, lines 1–4)

And because we stopped telling stories we had this huge gap—Black folk been catching babies. Black folk been doing abortion. Black folk been healing outside of a doctor’s office. Black folk been doing this, but we stopped talking about it. So, to start telling stories again and to share stories, that’s what’s going to heal our nation. That’s going to heal our people.^2^

Amber Nicole Thurman

Candi Miller

Porsha Ngumezi

Josseli Barnica

Neveah Crain

We say their names. They are the most recent casualties of the restrictive abortion bans enacted in the aftermath of *Dobbs v. Jackson Women’s Health Organization*,^3^ along with likely many others whose names are unknown. The common thread between these individuals is that their deaths were preventable.^4^ With this context, we assert the need to decenter medical spaces as the primary sites of care for women and birthing people. We assert this work requires an all-hands-on-deck approach that calls for the inclusion of liberatory approaches to reproductive care that affirm community-based and ancestral knowledge and do not subject our lives to the whims of political ideologies. Storytelling as a Reproductive Justice (RJ) method^5^ is a key site for resurrecting sacred knowledge that advances a critical dialogue on women’s and birthing people’s reproductive health choices.

The Afiya Center’s Livable Black Futures (LBF) Collective is a vital intervention born following COVID-19 and in response to the ongoing Black maternal mortality crisis.^6^ We are a collective of community-based doulas, advocates, and researchers working to build robust communities to confront reproductive injustices within our community. Following our formation of the collective in 2020 came the 2021 Texas abortion ban, SB-8, “The Heartbeat Bill.”^7^ Then, shortly after, the 2022 *Dobbs v. Jackson decision*.^3^ The accumulation of these legislative-based reproductive restrictions made our work even more urgent, and we knew that these setbacks would bear most harmfully on Black and Brown women and birthing people. We were and are seeing the devastating consequences of denying people reproductive health options in the medical space and witnessing the policing of the womb, as legal scholar Michelle Goodwin so aptly describes it. Goodwin writes: “Fetal protection and personhood efforts are not only on the rise in the United States, but like prior ‘tough on crime’ rhetoric, they serve as the bases on which to justify myriad unconstitutional and unethical interventions in women’s lives” (pp. 3–4).^8^ This policing has incredible costs, according to Goodwin—the rise of incarceration of pregnant women and birthing people and creating an atmosphere of distrust in the physician–patient relationship.^8^ More than that, it creates a conflict for medical providers who have essentially become representatives of the state, thereby limiting their ability to provide life-saving care to pregnant people.

The consistent fact underlying the deaths of Amber Thurman, Candi Miller, Porsha Ngumezi, Josseli Barnica, and Neveah Crain is that health care providers were afraid to provide care until it became apparent that the mother’s life was in danger,^4^ and by then, it was too late. In most of these cases, the mothers left behind children who cry for them in the night and who no longer have them in their lives. If the goal of pro-life is to value all life, then what of the lives of the children left behind? Dorothy Roberts’s *Killing the Black Body*^9^ catalogs the manifold assaults on Black reproduction and Black mothers as a means to extinguish Black families. It remains a North Star as we consider the collateral damaging effect of removing Black and Brown women from their families because of the state’s criminalization of bodily autonomy and the refusal of life-saving care. RJ is “the human right to maintain personal bodily autonomy, have children, not have children, and parent the children we have in safe and sustainable communities,”^5^ which remains key when considering the denial of these deceased individuals’ right to bodily autonomy and care and ability to be present mothers. RJ storytelling offers an embodied dimension to the issues we face. Sharing our stories is key to removing isolation and stigma.

Amber Thurman was a 28-year-old medical assistant in Georgia and mother to a 6-year-old boy who sought an abortion via mifepristone after an unexpected pregnancy.^4^ She died from the restrictive nature of the abortion related-ban, which contributed to the health staff’s reluctance to perform the medically necessary dilation and curettage procedure (D&C) that she desperately needed.

Candi Miller, a 41-year-old mother of 4 with lupus, diabetes, and hypertension, died because of a self-managed abortion because she knew that a pregnancy at her advanced maternal age would endanger her life, given her comorbidities. Because of fear of Georgia’s abortion ban, she died in isolation, afraid to seek care.^4^

Porsha Ngumezi, a 35-year-old woman from Texas, died from being denied a necessary D&C after complications from prescribed misoprostol taken to help her body pass tissue from a miscarriage.^4^

Twenty-eight-year-old Texas woman, Josseli Barnica, died from waiting 40 h for life-saving care from a miscarriage-in-progress at 17 weeks’ gestation.^4^

Neveah Crain, an 18-year-old Texas woman, died from an infection from a fetal demise that was also causing her to become ill.^4^ Doctors did not intervene until it was too late. These are the stories we know. There are so many more that we do not. What these stories show is that abortion care is not killing women and birthing people; abortion bans are.

The movement’s RJ godmothers, Loretta Ross and Marsha Jones, call for us to use storytelling to tap into other modes of healing and consciousness-raising in the face of attacks on bodily autonomy. Texas RJ godmother Marsha Jones reminds us, “Black folk been healing outside of a doctor’s office.”^2^ So, we are concerned with the inquiry: How can our current practice of RJ storytelling facilitate healing outside of the doctor’s office?

Our LBF storytelling-based circles conducted in 2021–2022 provide some answers. The RJ storytelling used in LBF is a methodology developed in our 2021–2022 LBF Storytelling research. We found that this form of RJ storytelling advances Black sexual and reproductive liberation narratives to address the silencing of voices regarding sexual and reproductive health. RJ storytelling in praxis can entail communal sessions as sites of destigmatization, knowledge production, and healing. It can also be enacted through singular testimonies, creative expression, and other narrative forms. We contend that this method as an intervention can disrupt silences, shame, and isolation—the oft-underexamined and undervalued factors that contribute to Black maternal mortality and morbidity. These factors were salient across many of the stories of those who have died from abortion bans. Our work found that RJ storytelling can serve as a site of ancestral reproductive wisdom and transgenerational healing that dismantles some of the driving factors leading to Black maternal death.

The LBF research collective gathered a Black multigenerational group (20–60 years old) where we drew on stories of ancestors and worked to undo confining narratives that produced shame. We also found that RJ storytelling goes beyond abortion stories, which do critical work to address the stigma attached to abortion. RJ storytelling is a key site for counterhegemonic reproductive knowledge. For example, before the *Dobbs v. Jackson* decision,^4^ we had been dealing with the precursor of the decision in the form of Texas’s “Heartbeat Bill,”^7^ which banned abortion after 6 weeks or when a fetal heartbeat is detected. Our discussions yielded significant insight into what people were contending with as they grappled with efforts to remove their reproductive freedom. One storyteller in LBF expressed: “They’re trying to put bans on our bodies.” These words carry forward the discourse of the Bans Off Our Bodies campaign led by Planned Parenthood^10^ in coalition with other organizations working to advance reproductive freedom. This work has become increasingly crucial as the encroachment on the bodily autonomy of women and birthing people is ever-increasing, from abortion bans to restrictions on gender-affirming care and more. Our LBF storytellers raised concerns about increasing costs to travel out of state for an abortion, the fear of criminalization, and having to turn to Crisis Pregnancy Centers^11^ with a history of deceptive practices, including overestimating a fetus’s weeks of gestation and projecting morals onto individuals seeking care. Additionally, regarding the Texas abortion bans, our LBF storytellers also expressed concern about what these developments would mean for the criminalization of Black women and birthing people who already navigated the criminalizing gaze of antiblackness and medical misogynoir.^12^

Most critically, RJ storytelling offers support for making informed reproductive health choices. We shared various insights through shame-free conversations on reproductive and sexual health. This critical work destigmatized reproductive health decisions and made members of our community feel less alone. We know legislation and litigation move slowly, particularly the legal changes we need to ensure reproductive health equity, such as unbiased access to reproductive health resources, opportunities, and outcomes, especially as it relates to abortion care. Thus, as an organization and scholars, we are advancing RJ storytelling to facilitate healing outside doctors’ offices in our community. This essay only sheds light on our inaugural storytelling circles; our LBF journey carries on as we continue this work, which includes the stories of Black gender- and sexually diverse birthing people, formerly incarcerated Black mothers, Black women aging while living with HIV (Note: to be sure, members of the communities above were in the inaugural experience as well), and the development of university courses training future RJ scholars on the importance of storytelling. We continue to fight for women’s and birthing people’s right to bodily autonomy to make whatever reproductive health decisions that they need, no matter the setting. The words of Josseli Barnica’s husband call attention to the urgency of these issues. He shares that the doctors told him that they had to “wait until there was no [fetal] heartbeat” before they could intervene to support Josseli.^9^ Sadly, the wait for a stopped fetal heartbeat cost Josseli Barnica her own. Medical providers should not have to make these kinds of deadly choices. In highlighting these stories, we utilize the work of RJ storytelling as a tool of advocacy and a reminder to all of us that we are not alone.

So now we ask you, in your community, what can RJ storytelling offer as a critical intervention space for knowledge-sharing, healing, and community building so that we do not continue to lose lives?

## Abbreviations Used

D&C: Dilation and curettage procedure
LBF: Livable Black Futures
RJ: Reproductive Justice
